# *ΔΔ*PT: a comprehensive toolbox for the analysis of protein motion

**DOI:** 10.1186/1471-2105-14-183

**Published:** 2013-06-07

**Authors:** Thomas L Rodgers, David Burnell, Phil D Townsend, Ehmke Pohl, Martin J Cann, Mark R Wilson, Tom CB McLeish

**Affiliations:** 1 Biophysical Sciences Institute, Durham University, Durham, UK; 2 Department of Chemistry, Durham University, Durham, UK; 3 School of Biological and Biomedical Sciences, Durham University, Durham, UK; 4 Department of Physics, Durham University, Durham, UK

## Abstract

**Background:**

Normal Mode Analysis is one of the most successful techniques for studying motions in proteins and macromolecules. It can provide information on the mechanism of protein functions, used to aid crystallography and NMR data reconstruction, and calculate protein free energies.

**Results:**

*ΔΔ*PT is a toolbox allowing calculation of elastic network models and principle component analysis. It allows the analysis of pdb files or trajectories taken from; Gromacs, Amber, and DL_POLY. As well as calculation of the normal modes it also allows comparison of the modes with experimental protein motion, variation of modes with mutation or ligand binding, and calculation of molecular dynamic entropies.

**Conclusions:**

This toolbox makes the respective tools available to a wide community of potential NMA users, and allows them unrivalled ability to analyse normal modes using a variety of techniques and current software.

## Background

Normal mode analysis (NMA) is both one of the most commonly used and best suited theoretical methods for studying motions in proteins and other macromolecules. This produces a collection of collective modes which represent the true protein dynamics [1]. The first normal mode studies were performed in the early 1980s [2-4], and they remained restricted to small-size proteins until the mid 1990s. From this time, methodological advances [5-9], simplified protein descriptions [10-13], and faster computer systems allowed them to address increasingly large macromolecular systems. By the early 2000s, entire protein complexes could be addressed, including the whole ribosome [14-16].

Krebs *et al.* 2002 [17] have analysed more than 3800 experimentally determined protein motions, and have shown that more than half of them can be approximated by applying a perturbation in the direction of at most two low-frequency normal modes of the considered protein; often a single low frequency normal mode is enough, and it is usually one of the three lowest-frequency modes [14,15]. Conformational changes on ligand binding of proteins have also been represented by motion along low frequency normal modes [8,14,15]. This method has also been used in the study of membrane channel opening [18], the analysis of structural movements of the ribosome [16], viral capsid maturation [19], transconformations of the SERCA1 Ca-ATPase [9,20], tertiary and quaternary conformational changes in aspartate transcarbamylase [21], mapping G-actin crystal form onto the F-actin crystal form highlighting possible transition pathways [22], the regulation of the Kv7.1 Potassium Channel by KCNE1 [23], and the unfolding of Amylosucrases [24].

B-factors calculated from crystallographic data have been predicted and refined using normal mode analysis [25,26]. The residue average B-factors (the average over all the heavy atoms, i.e. not including hydrogens) of alpha lytic protease have been well predicted [27] and extended to examine differences in motion of the S1 binding pocket in either a symmetric or antisymmetric direction. It has been found that the symmetric direction allowed a much large opening of the binding pocket. The diffuse scattering produced by correlated displacements of atoms during X-ray scattering experiments have also been predicted from normal mode analysis for lysozyme [28]. Cryo-EM structures have also been refined using elastic network models [29].

NMA is most often used to predict conformational changes that proteins undergo to fulfil their function, and can be used to check if a conformational change proposed on the basis of non-structural experimental data is likely to occur. These functional motions have led to the determination of domains within the proteins [30]. For example, Class I major histocompatibility complex molecule fluctuations have been found to be dependant on the conformation of their three domains [31] and it has been shown that each domain motion has a different function within the molecule. Human growth hormone induces dimerization of its binding protein; it has been shown that this is due to a marked decrease in domain motion after binding [32].

NMA can also be used to predict entropy changes on ligand binding as each normal mode has a calculable entropy associated with it. This means that for entropically controlled allosteric binding, it would be possible predict changes in the allosteric binding ratios [33]. The free energy of large functional motions can also be predicted by NMA [34,35]. The vibrational energy of G-actin has been calculated by regarding the molecule as a collection of independent harmonic oscillations (the normal modes) [35].

The major goal of normal mode analysis is to reduce the complexity of the full dynamics of a complex system and to describe them in a few generalised coordinates. However, if the long range hydrodynamics of water and anharmonicity are important variants to the protein motion then a method that is capable of reducing a complex system to a few general components but is not dependant on a harmonic approximation is needed. This method is principle component analysis (PCA) [36], and it is a technique used in a wide variety of fields, e.g. from finance to biology.

PCA computes the second moment of a multivariate distribution and describes the deviations from an average in terms of a set of principle components that represent the collective motions of the largest deviations. These principle components are the eigenvectors of a covariance matrix of the motion, whether the system is harmonic, heavily damped, or does not oscillate at all. Like NMA generally, only a small number of the lowest frequency modes are needed to describe most of the protein motion [37]. The lowest frequency modes tend to describe possible conformational changes in the protein while the slightly higher frequency modes describe vibrational, or breathing, motions around the average structure.

For ubiquitin, with molecular dynamics simulations starting from a variety of different X-ray structures, it was found that the first ten quasi-harmonic analysis modes contributed 78% of all the dynamic movement and that these modes described fluctuations of the structures seen with NMR [38].

PCA need not even be applied to dynamic fluctuations, but can be used to explore a mapping of many different conformers or mutants of a family of proteins. Recent work has explored 40 different X-ray structures of Ras kinase proteins and found that the structural variance can be described by a small number of principal components [39].

NMA and PCA thus represent a powerful tools with a wide range of applications in structural biology. Due to this there are a number of on-line web servers currently available that can calculate elastic network models, e.g. EL-Nemo [15] provides the scaled frequency, fluctuations, and shapes of calculated normal modes; ANM web server [40] provides calculation of the normal modes and allows on-line display of the modes with a Jmol plugin; and FlexServ [41] provides calculation of normal modes, and also allows simulation by discrete molecular dynamics and Brownian dynamics.

There are also programmatic libraries available for analysis of NMA and PCA, however, these mean the user has to write their own code and integrate the subroutines from these libraries manually; e.g. MMTK [42] which provides python subroutines for molecular dynamics, NMA, and structural minimisation; and ProDY [43] which provides python subroutines for PCA and NMA.

We designed *ΔΔ*PT as a comprehensive, but still easy-to-use toolbox for NMA/PCA, with increased functionality for normal mode analysis over currently available methods, and easier to use than current programmatic libraries. Particular emphasis was put on its ability to analyse data from Elastic Network Models, Gromacs simulations [44], Amber simulations [45], and DL_POLY simulations [46,47] in an interchangeable manner with all the post analysis tools available irrespective of the input data. Due to the modular nature of the software it is also easy to produce additional input or analysis programs to adapt to the needs of most researchers; however, this is not required to use the program.

## Methodology

Normal mode calculation is based on the harmonic approximation of the potential energy function, *V*, around a minimum energy conformation, Equation 1, where *r* is the distance between atoms, *R* is the equilibrium distance between atoms, *u* is the difference from equilibrium distance between atoms, *i* and *j* refer to the atom number, and *α* and *β* refer to the direction of the motion.

(1) $$V\left(r_{n}\right)=\frac{1}{2}\underset{\alpha =x,y,z}{\overset{N}{\underset{i}{\sum }}}\underset{\beta =x,y,z}{\overset{N}{\underset{j}{\sum }}}{\left(\frac{\partial^{2}V}{\partial r_{\mathrm{i\alpha}}\partial r_{\mathrm{j\beta}}}\right|}_{R}u_{\mathrm{i\alpha}}u_{\mathrm{j\beta}}$$

This approximation allows an analytic solution of the equations of motion by diagonalising the mass-weighted Hessian matrix, **D**, (the mass-weighted second derivatives of the potential energy matrix), Equation 2, where $D_{\mathrm{i\alpha},\mathrm{j\beta}}={\left(\partial^{2}V/\partial r_{\mathrm{i\alpha}}\sqrt{m_{i}}\partial r_{\mathrm{j\beta}}\sqrt{m_{j}}\right|}_{R}$ and *m* is the mass.

(2) $$e^{-1}De=\text{diag}\left[\omega_{1\to n}^{2}\right]$$

The eigenvectors of this matrix, *e*, are the normal modes, and the eigenvalues are the squares of the associated frequencies, *ω*. The protein movement can then be represented as a superposition of these normal modes, fluctuating around a minimum energy conformation. The normal modes responsible for most of the amplitude of the atomic displacement are associated with the lowest frequencies.

In order to avoid time-consuming energy minimisations, a single-parameter Hookean potential can be used, which is shown to yield low-frequency normal modes as accurate as those obtained with more detailed, empirical, force fields [10]. The spring constant of the Hookean potential, *k*, is generally assumed to be the same for all interacting pairs within an arbitrary cut-off, *R*_*c*_, beyond which interactions are not taken into account.

(3) $$V_{\text{ij}}=\left\{\begin{array}{cc} \frac{k_{\text{ij}}}{2}{\left(r_{\text{ij}}-R_{\text{ij}}\right)}^{2} & \;R_{\text{ij}}^{2}\leq R_{c}^{2}\\ 0 & \;R_{\text{ij}}^{2}>R_{c}^{2} \end{array}\right)$$

**Figure 1:**
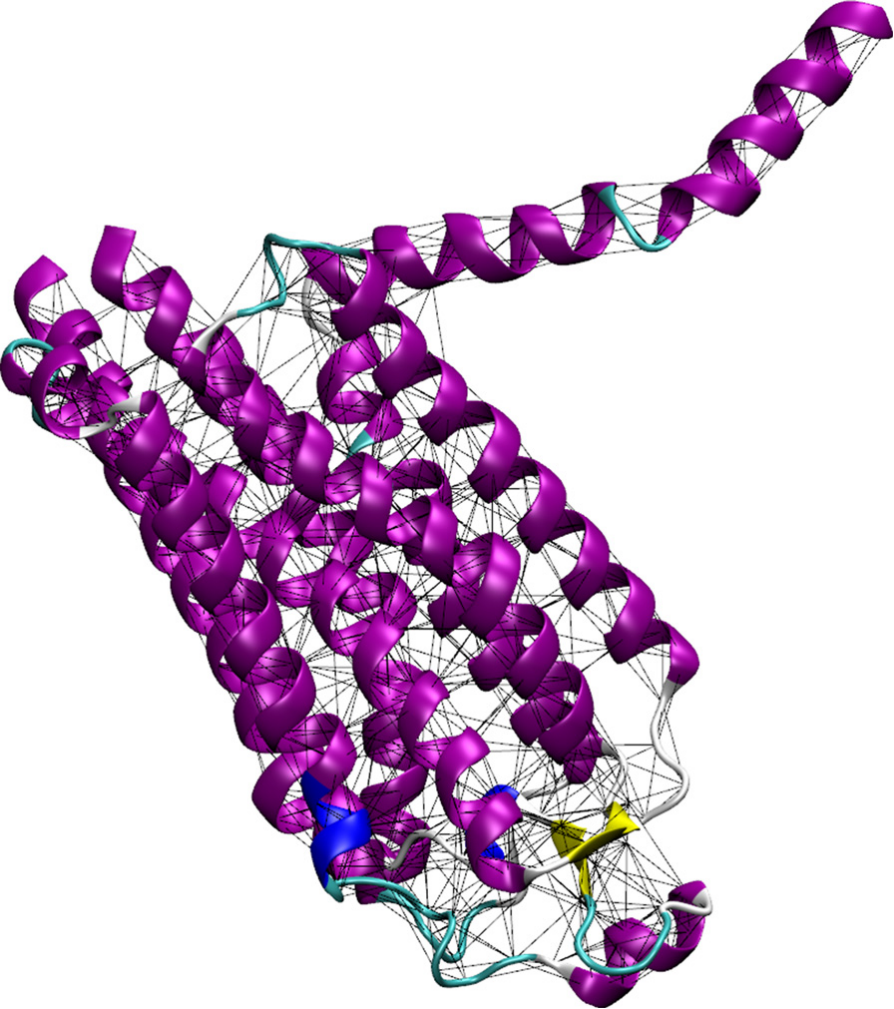
**Example ENM springs with a cut-off of 8 Å.** Example ENM springs with a cut-off of 8 Å for Adenosine A2a receptor (pdb: 2YDV, [48]). Colours correspond to the secondary structure of the protein assigned by STRIDE [49]; regions defined as alpha helix are coloured purple, regions defined as beta sheets are coloured yellow, turn regions are coloured cyan, and coil regions are coloured white.

*ΔΔ*PT toolbox has a default cut-off of 12 Å and a Hookean potential of 1 kcal mol ^-1^ Å ^-2^, these can be changed with the relevant flags (-c and -r respectively) in the GENENMM program. This approximation implies that the reference structure represents the minimum energy conformation. As default, all atom masses are set to the same fixed value in the kinetic energy term, 1 Da, as this approximation was shown to have little influence on the low-frequency modes; however, if desired the true atomic masses can be used (add -mass flag) or, if the model is based on the C_*α*_ atoms, only the residue mass can be assigned (add -res flag with -ca flag), Figure 1.

The GENENMM program also allows elastic network models with a varying spring constant, either with an empirical power decay on the interaction (-an flag), with the Hinsen exponential spring constant (-hine) [12], with Hinsen fitted spring constants (-hin) [42], or with individually set values between residues (-f *file*). GNMPROD also allows the production of the one-dimensional Gaussian network model instead of the three-dimensional elastic network model^a^.

The resulting Hessian can be either fully diagonalised using the DIAGSTD program (not recommended for many more than 1000 sites - although in reality a system this size will only take around 10 minutes to solve on a desktop PC - run serial on an AMD Phenom™II 3.2 GHz Quad Core) or diagonalised using the rotation-translation-block (RTB) approach, DIAGRTB program. The RTB approach groups several atoms into a single point, which is generally achieved by division into residue blocks, or multiple residue blocks. The rigid-body rotations and translations of these ‘super’-sites are used as the new co-ordinate system instead of Cartesian co-ordinates [6]. When a small number of residues per block are used, the approximation has very little effect on the low frequency modes; although the frequencies do increase predictably due to internal block stiffening [8]. Using this approximation, it becomes possible to treat very large proteins, or protein complexes, in an all-atom level of description in reasonable computing time. DIAGRTB can be set to block into groups by a number of residues (-r *n*), block into the protein secondary structure (-str SECO), or block into custom domains (-str DOMN). The lowest frequency modes mainly depend on the overall shape of the system; they can be captured at extremely high levels of coarse-graining [50] or by using low-resolution structural data [51].

For comparison with atomistic simulations, the COVAR program allows calculation of a mass weighted covariance matrix, **F**, from trajectories generated with Gromacs, Amber, or DL_POLY, Equation 4, where **x** is the atomic position matrix and **m** is the mass matrix.

(4) $$F=\langle m^{1/2}\left(x-\langle x\rangle \right){\left(m^{1/2}\left(x-\langle x\rangle \right)\right)}^{T}\rangle$$

COVAR also corrects the displacements by removing the centre of mass motion and rigid body rotations; this produces more accurate results as the motion is not dominated by the rigid body motions. These displacements can be expanded into normal modes, principle component analysis, Equation 5, where *Q* is the eigenvalue matrix.

(5) $$e^{-1}Fe=\langle Q^{2}\rangle$$

As we are again approximating the full motion to harmonic style motions, the solution is governed by harmonic oscillatory statistical mechanics. This means that for each eigenvector Equation 6 must hold true [52], where *k* is the Boltzmann constant, *T* is the temperature, and *v* is the normal mode number.

(6) $$\langle Q_{v}^{2}\rangle =\frac{\text{kT}}{\omega_{v}^{2}}$$

**Figure 2:**
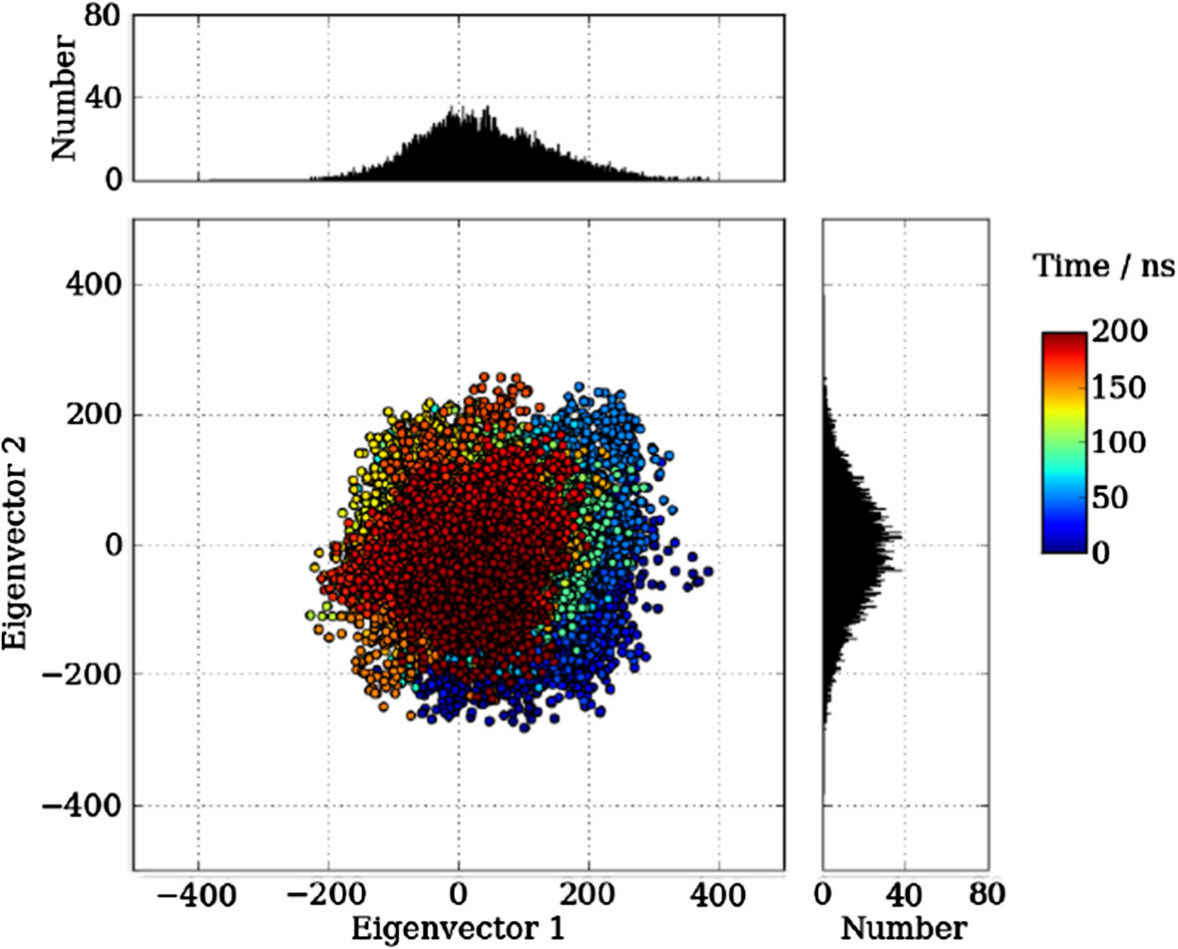
**Plot of the trajectory from an Amber simulation.** Plot of the trajectory from an Amber simulation of CAP (pdb: 1G6N) onto the two lowest frequency eigenvectors. Each trajectory position is plotted as the dot product of the co-ordinates and the eigenvector, representing the extend of the displacement along each eigenvector from the average position. The distribution of these values are displayed as the adjoining histograms. The colour of the points responds to the simulation time.

The COVAR program also plots the trajectory frames onto the lowest frequency eigenvectors, Figure 2. If the inbuilt principle component analysis tools in Gromacs or Amber are preferred, GroAMED can convert the default outputs respectively for use of the other toolbox programs.

The FREQ/EN program calculates the mode frequencies, the free energy, and the entropy from the calculated eigenvalues. The free energy and entropy are calculated using the full solution, Equations 7 and 8, and the Schlitter approximation [53] for comparison with other programs^b^, where *G* is the free energy, *S* is the entropy, and ℏ is the reduced Plank constant.

(7) $$G_{v}=-\text{kT}\ln\left(\frac{1}{1-\text{exp}\left(-\frac{\hbar \omega_{v}}{\text{kT}}\right)}\right)$$

(8) $$S_{v}=k\left(\frac{\frac{\hbar \omega_{v}}{\text{kT}}}{\text{exp}\left(\frac{\hbar \omega_{v}}{\text{kT}}\right)-1}-\ln\left(1-\text{exp}\left(-\frac{\hbar \omega_{v}}{\text{kT}}\right)\right)\right)$$

The RMS/COLL program calculates the root mean squared displacements of all the atoms for each of the selected modes along with the collectivity, *κ*, of the modes, Equation 9 [54], where *α* is the collectivity constant selected so that $\overset{N}{\underset{i}{\sum }}\alpha \left|e_{i}^{2}(v)\right|=1$ and *N* is the number of atoms.

(9) $$\kappa_{v}=\frac{1}{N}\text{exp}\left(-\overset{N}{\underset{i}{\sum }}\alpha \left|\overset{2}{\underset{i}{e}}(v)\right|\text{log}\left(\alpha \left|\overset{2}{\underset{i}{e}}(v)\right|\right)\right)$$

The degree of collectivity indicates the fraction of atoms that are significantly affected by a given mode. For modes involving all the atoms, the degree of collectivity tends to be one, whereas for localised motions the degree of collectivity approaches zero (actually 1/*N*). The first 25-50 low-frequency normal modes tend to have a collectivity of above 0.4 meaning a significant part of the protein is involved in each mode. Low collectivity in the lowest frequency modes is indicative of extended parts of the system, either N- or C- termini or large unstructured loops. These loops cannot be modelled in a meaningful way as they intrinsically adopt multiple conformations, can appear to be invisible in one crystal form but visible in a different crystal form [55], or can even appear ordered due to crystal packing [56].

It is common practise to remove these extended parts prior to the normal mode computation. If a RTB approximation is used, there is some advantage to blocking and representing large unstructured loops by one block so they are included but do not dominate the motion.

The RMS/COLL program also calculates the B-factors, *B*, Equation 10 [52], from the mean square displacements of the first 25 lowest frequency modes (this can be changed with the -e *n* option), ignoring the six rigid block rotational and translation modes (starting from the seventh mode, -s 7, is the default). The B-factors should be calculated with the same mass weighting options as GenENMM. Correlations to crystallographic B-factors are typically found to be greater than 0.5-0.6 [15], and can even be greater than 0.8 [1]^c^.

(10) $$B_{i}=\frac{8\text{kT}\pi^{2}}{3m_{i}}\underset{v}{\sum }\frac{\left|e_{i}^{2}(v)\right|}{\omega_{v}^{2}}$$

Adjusting the cutoff value can slightly improve such correlations, and if possible it is recommended that the correlation between the shape of the predicted and the experimental values is iterated upon when setting the cutoff value if no other information is available. The comparison between the shape of the computed and observed crystallographic B-factors provides a measure of how well the protein’s flexibility in its crystal environment is described by the normal modes. This motion tends to echo, but is more restricted (by crystal packing) than the motion in solution.

**Figure 3:**
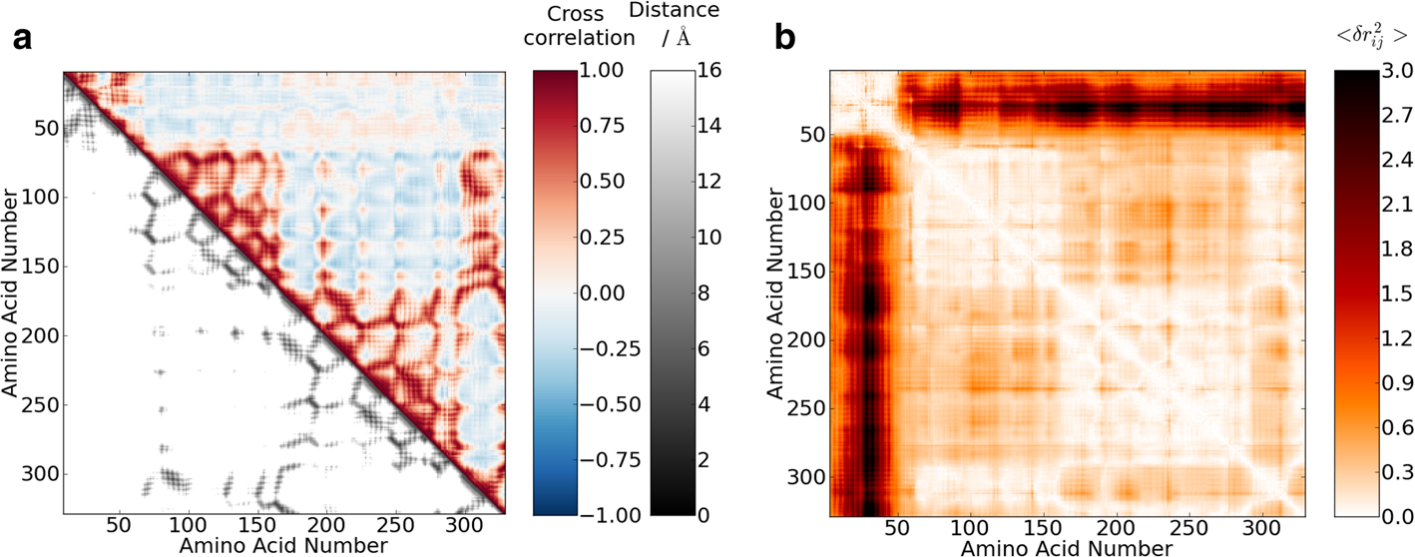
**Cross correlation and mean square fluctuations.** Plots of (**a**) the cross correlation of the residue motions and the distance between the C_*α*_ atoms, and (**b**) the mean square fluctuations of each residue for LAC (pdb: 1EFA). The cross correlation of the C_*α*_ atom motion is calculated from Equation 11 which defines how similar the motion direction is, 1 is identical motion, 0 is completely different motion, while -1 is exactly inverse motion. The mean square fluctuations of each residue is calculated from Equation 12 and represents how much the distance between each residue varies during the natural protein motion.

The CROSCOR program calculates the cross-correlation, *C*, of atoms over the first 25 modes (although this can be changed with the -b *n* and -e *n* flags), Equation 11. The cross-correlation shows which atoms tend to move in the same direction with a correlated motion in the modes, Figure 3(a). A value of 1 implies perfectly correlated motion and -1 perfectly anti-correlated motion. As the numerator is calculated as the dot product between the two vectors, as is a common manner of calculation, the correlation is dependant on the angle of the motion, i.e. fluctuations of the same period and phase but with a difference in orientation of 90° will give a value of 0. Thus, the cross-correlation is useful for identifying which atoms make up a group with correlated motions; however, a spherical breathing mode is difficult to identify from the cross-correlations because they are positive for atoms on the same side, negative for atoms on opposite sides, and 0 for atoms at 90° [57].

(11) $$C_{\text{ij}}=\underset{v}{\sum }\left(\frac{e_{i}(v)\cdot e_{j}(v)}{{\left({\left|e_{i}(v)\right|}^{2}{\left|e_{j}(v)\right|}^{2}\right)}^{0.5}}\right)$$

The extent of this motion, $\langle \delta r_{\text{ij}}^{2}\rangle$, can be calculated with the MOVEING program. This calculates the change in the distance between atoms between the equilibrium value and the value after applying the eigenvectors, Equation 12, Figure 3(b).

(12) $$\;\langle \delta r_{\text{ij}}^{2}\rangle =\underset{v}{\sum }\left(\frac{{\left(x_{i}+e_{i}(v)-\left(x_{j}+e_{j}(v)\right)\right)}^{2}-{\left(x_{i}-x_{j}\right)}^{2}}{\omega_{v}^{2}}\right)$$

The OVERLAP program calculates the overlap of the atomic motion between eigenvectors, *v*_1_ and *v*_2_, Equation 13. There can be different eigenvectors for the same NMA, eigenvectors produced with ENM to atomistic NMA, NMA eigenvector to difference in two crystal structures, or any combination thereof. A values of 1 indicates that the motions are identical whereas a value of 0 indicates that the motions are completely different.

(13) $$I_{v_{1}v_{2}}=\left(\frac{\underset{i}{\sum }\left|e_{i}(v_{1})\cdot e_{i}(v_{2})\right|}{{\left(\underset{i}{\sum }{\left|e_{i}(v_{1})\right|}^{2}\underset{i}{\sum }{\left|e_{i}(v_{2})\right|}^{2}\right)}^{0.5}}\right)$$

## Implementation

There are four principal inputs into the toolbox: protein coordinates written in PBD format [58]; NMA output data from Gromacs; PCA output data from Gromacs or Amber; or a trajectory output from Gromacs, Amber, or DL_POLY.

For the ENM implementation, the PDB file where all ATOM records are read by the GENENMM program is all that is needed to determine the interaction matrix (HETATM records can also be included by using the -het flag, these are commonly used for ligands and provide an easy method of looking at differences on ligand binding). DNA can also be read into the GENENMM using the -DNA flag; this then includes the C4 and C1’ carbon atoms if the -ca flag (for C_*α*_ only) is used. This interaction matrix is output so that it can be solved directly using either DIAGSTG or blocked in RTBs and solved with DIAGRTB. This simple approach will likely produce useful results when using an original (unprocessed) PDB file, but some modifications of the input data are advisable, e.g. removal of water or buffer molecules. To prevent lumping of residues that are part of separate molecules into one RTB residue, different chain identifiers should be used. Alternate amino-acid conformations should be removed (if present) and hydrogen atoms should be erased, as their presence will have only a minor influence on the results but a large effect on the solution time (using the -ca flag will automatically ignore any atoms that are not C_*α*_ atoms).

After solving the interaction matrix or covariance matrix from a simulation, the eigenvalues and eigenvectors will be output into a single file. These can be analysed with any of the tools mentioned and the normal modes can be conveniently viewed with the NMWIZ plugin for VMD [43]. The NMWIZWT tool will convert the calculated values into the relevant input file for the NMWIZ plugin.

**Figure 4:**
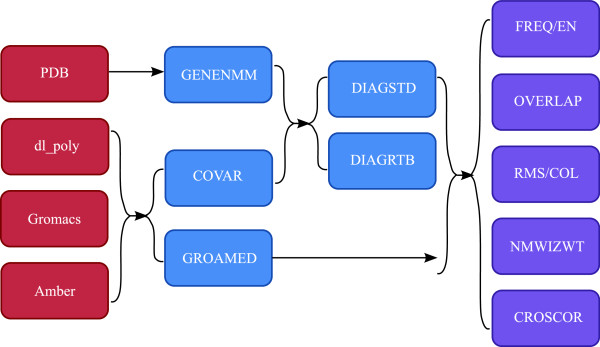
**Minimal flow sheet for *Δ Δ *PT.** Minimal flow sheet for *ΔΔ*PT. Red boxes are the types of input files which can be used with *ΔΔ*PT, blue boxes are the main processing programs, while red boxes are the subsequent analysis programs provided by *ΔΔ*PT.

Table 1 contains a list of, and a brief description of, the programs included in the *ΔΔ*PT toolbox; Figure 4 shows a minimal flow sheet for *ΔΔ*PT.

**Table 1:** ***Δ Δ *PT tools**

**Tool**	**Description**
G E N E N M M	Generates interaction matrix for an ENM
D I A G S T D	Diagonalises an interaction matrix
D I A G R T B	Diagonalises an interaction matrix with the RTB approximation
D O M A I N S	Rewrites pdb into custom domain order for domain RTB
G N M P R O D	Calculates the normal modes for a GNM
F R E Q / E N	Calculates the frequencies and energies for a set of normal modes
R M S / C O L	Calculates the motion, B-factors and collectivity for a set of normal modes
C R O S C O R	Calculates the cross correlation for a set of normal modes
O V E R L A P	Calculates the overlap of a set of normal modes
G R O A M E D	Converts Gromacs and Amber output for use with the toolbox
C O V A R	PCA decomposition for Gromacs, Amber, or DL_POLY trajectories
F U L L 2 C A	Reduces outputs for full protein into C_*α*_ only outputs
T R A J P D B	Converts trajectories from Gromacs, Amber, or DL_POLY into a pbc fixed pdb file
N M W I Z W T	Produces input for the nmwiz plugin for VMD
P R O J E C T	Produces a set of pdb files for each normal mode perturbed from the input structure
E G N P R O J	Plots the trajectory frames onto the eigenvector space
P D B D I F F	Produces the vector between two pdb structures
S P A C I N G	Calculates the inter-atom distances for plotting
M O V E I N G	Calculates the change in atom positions due to the normal modes
P L O T P D B	Writes a file of residue based values onto a pdb for plotting in VMD

## Conclusions

NMA is a powerful tool for the study of protein movements, conformational changes, and protein entropy. It compliments experimental techniques such as X-ray crystallography and NMR, has been used extensively in identifying different structural biology domains, and provides new insights into entropy changes on binding.

This toolbox has increased functionality over those of the currently available web servers, e.g. EL-Nemo [15] and ANM web server [40]. Its main advantages are its abilities to provide the user with tools for analysing elastic network models and molecular dynamics simulations, and for users to add their own extra modules and functions if needed.

This toolbox makes the respective tools available to a wide community of potential NMA users, and allows them unrivalled ability to analyse normal modes using a variety of techniques and current software. With consistent file types, information can be easily exchanged and compared between methods. The availability of a comprehensive and easy-to-use dedicated NMA downloadable software will therefore facilitate further research into this interesting technique.

## Availability and requirements

**Project name:***ΔΔPT***Project home page:**https://sourceforge.net/projects/durham-ddpt/**Operating system(s):** Platform independent**Programming language:** fortran90**Other requirements:** gfortran 4.4.1 or higher, or ifort 11.1 or higher**License:** GNU GPL**Any restrictions to use by non-academics:** none

## Nomenclature

Roman

*B* B-factor -*C* Cross correlation - **D** Mass weighted Hessian matrix J mol ^-1^ m ^-2^ Da ^-1^ **e** Eigenvector m Da ^-1^ **F** Mass weighted covariance matrix Da m^2^*G* Free energy J mol ^-1^ℏ Reduced Plank constant 1.05457148×10^-34^ m^2^ kg s ^-1^*I* Overlap -*i* Atom number -*i* Atom number -*i* Atom number -*k* Boltzmann constant 8.314 J mol^-1^ K^-1^*k*_*ij*_ Hookean spring constant J mol ^-1^ m ^-2^*m* Mass Da*N* Number of atoms -*Q* Eigenvalue matrix -*R* Equilibrium distance between atoms m*r* Distance between atoms m*S* Entropy J mol ^-1^ K ^-1^*T* Temperature K*u* Difference from equilibrium distance between atoms m*V* Potential Energy J mol ^-1^*v* Normal mode number - x Atomic position matrix m**Greek***α* Collectivity constant - *α* Direction - *β* Direction - *κ* Collectivity - *ω* Eigenfrequency *s*^-1^

## Endnotes

^a^The Gaussian network model is explicitly represented as $V=\frac{K}{2}\overset{N}{\underset{i,j}{\sum }}\left(r_{j}\Gamma_{i,j}r_{i}\right)$[11], where **r** is the distance between sites and *Γ* is the Kirchhoff matrix.^b^Note that for the ENM, there are always six frequencies that are several orders of magnitude lower than the others (the eigenvalues of these are essentially zero), these correspond to six solid block rotational and translational modes. If more than six very low frequency normal modes are obtained, this means that a group of atoms is at a distance larger that the cut-off radius from the other atoms.^c^The B-factors calculated by Equation 10 give only the contribution of the thermal fluctuations while the experimental B-factors also contain contributions from factors [59].

## Competing interests

The authors declare that they have no competing interests.

## Authors’ contributions

TLR wrote the paper. TLR and DB wrote the toolbox. All authors contributed to the design of the toolbox and substantially edited the manuscript. All authors read and approved the final manuscript.
